# Novel Nanoparticle-Based Cancer Treatment, Effectively Inhibits Lung Metastases and Improves Survival in a Murine Breast Cancer Model

**DOI:** 10.3389/fonc.2021.761045

**Published:** 2021-11-05

**Authors:** Sarah Kraus, Raz Khandadash, Raphael Hof, Abraham Nyska, Ekaterina Sigalov, Moshe Eltanani, Pazit Rukenstein, Ricarina Rabinovitz, Rana Kassem, Adam Antebi, Ofer Shalev, Moshe Cohen-Erner, Glenwood Goss, Arnoldo Cyjon

**Affiliations:** ^1^ New Phase Ltd., Petah Tikva, Israel; ^2^ Toxicologic Pathology, Sackler Faculty of Medicine, Tel Aviv University, Tel Aviv, Israel; ^3^ Division of Medical Oncology, University of Ottawa, Ottawa, ON, Canada; ^4^ Department of Oncology, Shamir Medical Center, Zerifin, Israel

**Keywords:** metastatic breast cancer (BC), alternating magnetic field (AMF), enhanced permeability and retention (EPR) effect, magnetic hyperthermia (MHT), iron oxide nanoparticles

## Abstract

Sarah Nanoparticles (SaNPs) are unique multicore iron oxide-based nanoparticles, developed for the treatment of advanced cancer, following standard care, through the selective delivery of thermal energy to malignant cells upon exposure to an alternating magnetic field. For their therapeutic effect, SaNPs need to accumulate in the tumor. Since the potential accumulation and associated toxicity in normal tissues are an important risk consideration, biodistribution and toxicity were assessed in naïve BALB/c mice. Therapeutic efficacy and the effect on survival were investigated in the 4T1 murine model of metastatic breast cancer. Toxicity evaluation at various timepoints did not reveal any abnormal clinical signs, evidence of alterations in organ function, nor histopathologic adverse target organ toxicity, even after a follow up period of 25 weeks, confirming the safety of SaNP use. The biodistribution evaluation, following SaNP administration, indicated that SaNPs accumulate mainly in the liver and spleen. A comprehensive pharmacokinetics evaluation, demonstrated that the total percentage of SaNPs that accumulated in the blood and vital organs was ~78%, 46%, and 36% after 4, 13, and 25 weeks, respectively, suggesting a time-dependent clearance from the body. Efficacy studies in mice bearing 4T1 metastatic tumors revealed a 49.6% and 70% reduction in the number of lung metastases and their relative size, respectively, in treated *vs*. control mice, accompanied by a decrease in tumor cell viability in response to treatment. Moreover, SaNP treatment followed by alternating magnetic field exposure significantly improved the survival rate of treated mice compared to the controls. The median survival time was 29 ± 3.8 days in the treated group *vs*. 21.6 ± 4.9 days in the control, *p*-value 0.029. These assessments open new avenues for generating SaNPs and alternating magnetic field application as a potential novel therapeutic modality for metastatic cancer patients.

## Introduction

Cancer is a major public health problem worldwide and is the leading cause of death in the Western world with a resultant significant detrimental economic impact (1). Current therapies, particularly in the metastatic setting are limited and better treatments are needed. Recent improvement in nanomaterials and the rapid development of nanotechnology provide an opportunity for new therapeutic strategies against cancer. Nanoparticles are particularly promising due to their good biocompatibility, based on their particle size, shape, and physicochemical properties (2). A rapidly growing body of literature has provided evidence suggesting a major role for nanotechnology in cancer treatment.

A variety of nanoparticles have been investigated as drug carriers, photothermal agents, contrast agents, and radiosensitizers (3). Of particular interest are their unique chemical properties, including the ability to bind amine and thiol groups, allowing surface modification and use in biomedical applications.

Physiologically, elevated body temperature can damage and kill cancer cells with minimal injury to normal cells, thereby providing a therapeutic index which can be exploited. Hyperthermia, the process of raising the temperature of tumor-loaded tissue to 40-43°C results in the denaturation of proteins and structural damage within cancer cells (4). During the last decades, hyperthermia based-cancer treatment has been applied safely mainly as an adjuvant therapy (5).

We have developed a novel nanoparticle-based treatment termed Sarah Nanotechnology system that selectively destroys cancer cells by hyperthermia. Sarah Nanotechnology comprises of: (i) Sarah Nanoparticles (SaNPs), containing encapsulated iron oxide (IO) nanoparticles, that attach to cancer cells, and an (ii) electromagnetic induction system (EIS) that generates an alternating current magnetic field (AMF) that is converted to heat by the SaNPs due to their magnetic properties. The applied AMF heats the SaNPs to a predefined and controllable temperature, thereby offering an innovative approach to treat cancer by inducing non-ablative thermic damage (5).

SaNP consists of 25 nm IO nanoparticles, a phase change material (PCM) core, and an encapsulating hydrophilic polymer comprised of amine functionalized 6-arm-branched polyethylene glycol (PEG) 20,000, that enables flexibility, increases biocompatibility, and significantly masks the SaNP from the body’s mononuclear phagocyte system (MPS) thereby reducing uptake by phagocytic cells (6). The PCM has a high energy storage capacity (latent heat). It can absorb, store or release large latent heat over a defined temperature range while the phase change occurs from solid to liquid and vice versa. Therefore, its capability of releasing, retaining, and absorbing latent heat energy during the phase transition allows for the storage of heat energy and thermal control (7). These principles were applied in the SaNP design. SaNP synthesis is detailed elsewhere (8).

SaNPs are intravenously (IV) administered to the patient and localize on cancer cells. They accumulate on cancer tissue at higher concentrations compared to normal tissue mainly due to the enhanced permeability and retention (EPR) effect, a central mechanism for passive tumor targeting, which allows extravasation of the nanoparticles and enables the preferential retention of SaNPs in tumors due to their leaky vasculature and reduced lymphatic drainage (9, 10).

Following delivery and attachment of the SaNPs to the malignant cells, the patient undergoes regional 290 ± 10% kHz AMF application with the EIS generating a magnetic field at a range of 8-33 kA/m, the SaNPs subsequently convert the applied AMF to latent heat and retain it, and the PCM core controls and stabilizes the SaNP temperature to 50 ± 3°C, thereby using this heat to cause hyperthermic cancer cell death without harming healthy cells under a magnetic field. The main innovation of the SaNP lies in its inherent ability to control its temperature without inducing thermal ablation which occurs at temperatures above 60°C (5). Previous studies examining the PCM’s functionality and the temperature control property have demonstrated that SaNPs exposed to AMF irradiation with an amplitude of 33.4 kA/m at 300 kHz for 30 minutes stabilize at a temperature plateau between 48–52°C, in agreement with the melting phase transition temperature range of the PCM component (8). Previous studies also showed no damage to healthy cells (8).

The 4T1 triple negative mammary carcinoma is a transplantable tumor cell line that can be grown *in vivo* as a primary tumor in BALB/c mice (11). Its major advantage is that 4T1 cells spontaneously metastasizes in a pattern that is analogous to human mammary cancer. When intravenously injected, 4T1 cells are capable of metastasizing to different organs characteristic of breast cancer, but predominantly to the lungs thereby mimicking breast cancer lung colonization and distal metastasis (12). Although the IV model is a commonly used murine model to study breast cancer lung metastasis, it may not accurately reflect human metastatic breast cancer as it does not follow the biological steps that a primary tumor must take to produce distant metastatic tumors. However, using genome-wide gene expression microarrays, it has been reported that there are no differences between metastatic lesions produced by IV injection of 4T1 cells compared to orthotopic implantation of the same cancer cells and the lung metastases have similar genetic profiles (13). Therefore, the model fairly represents the development of distant lung metastases while offering a faster progression without the need to remove the primary tumor which is often required in an orthotopic model due to tumor burden.

The current study shows the biodistribution of SaNPs in mice and the safety of treatment. Our preclinical animal cancer model studies demonstrate the *in vivo* therapeutic efficacy of Sarah Nanotechnology in treating BALB/c mice bearing 4T1 breast cancer lung metastatic tumors and the effect of treatment on prolonging survival.

## Materials and Methods

### Materials

Commercially available chemicals and reagents included the following:

Phosphate buffered saline (PBS, pH 7.4) was purchased from Sigma Aldrich, Rehovot, Israel.

Hank’s Balanced Salt Solution (HBSS), Fetal Bovine Serum (FBS), RPMI 1640 medium, 0.25% Trypsin-EDTA solution, sodium pyruvate, glutamine, and antibiotics (penicillin, streptomycin) were all from Biological Industries, Beit-Haemek, Israel. Puromycin was from Santa Cruz Biotechnology, Inc., TX, USA.

Amine functionalized 6-arm-branched, polyethylene glycol (PEG), 20 kDa molecular weight, was purchased from SunBio, South Korea. Water for injection (WFI) was from B. Braun Medical Inc., PA, USA.

### SaNP Characterization

SaNPs were characterized by dynamic light scattering (DLS) and transmission electron microscope (TEM) imaging to determine their physicochemical parameters and morphology.

Hydrodynamic size, size distribution, and zeta potential measurements were conducted using a Zetasizer Nano Series ZS (Nano-ZS, Malvern Instrument Ltd., UK) operating with a 4 mW HeNe laser (632.8 nm), a detector positioned at a scattering angle of 173°, and a temperature-controlled jacket for the cuvette. Three measurements consisting of up to 12 consecutive sub-runs were performed for each sample. Dynamic correlation functions were fitted by a second-order cumulant method to obtain the size distributions. For the zeta potential measurements, 0.8 mL of the SaNP dispersion were loaded into folded capillary zeta potential cells with integrated gold electrodes. Three measurements consisting of 12 sub-runs were performed at 25°C.

Samples for TEM were prepared by spreading 5 µl of the SaNP dispersion onto the surface of a 400-mesh carbon-coated copper grid (Electron Microscopy Sciences, USA). Uranyl acetate was used as the staining agent. The samples were air-dried at room temperature, and low-resolution TEM images were obtained using a Tecnai G2Spirit Twin T-12 electron microscope (Bar Ilan University, Israel).

### Cell Culture

The 4T1 mouse mammary carcinoma cell line was obtained from the American Type Culture Collection (Rockville, MD, USA). Cells were grown in RPMI 1640 medium supplemented with 10% (v/v) FBS, 1.0 mM sodium pyruvate, 100 U/mL penicillin, and 100 μg/mL streptomycin. Cells were maintained in a humidified atmosphere containing 5% CO_2_ at 37°C.

For imaging purposes, modified human embryonic kidney GP2-293 cells were co-transfected with pRetroQ-mCherry-N1 Vector using the complementary Retro-X™ Universal system (Clontech, CA, USA) to generate mCherry containing viral particles. pRetroQ-mCherry-N1 retroviral particles containing supernatant were collected 48hrs after transfection. 4T1 cells were infected and mCherry positive cells were selected by Puromycin (2µg/mL) resistance.

To generate a metastatic lung cancer model, mouse 4T1 mCherry breast cancer cells were grown to 70% confluency and metastatic tumors were established by harvesting early passage 4T1 cells with 0.25% Trypsin-EDTA, centrifuged at 500×g for 5 min, and resuspended in ice cold HBSS at 2.5×10^4^ cells/200μl solution. The cell suspension was IV injected via the lateral tail vein of BALB/c mice.

### Animals

BALB/c mice, 7-8 weeks old, were purchased from Envigo (Ness Ziona, Israel). All animal experiments were reviewed and approved by an Institutional Animal Care and Use Committee (IACUC), followed officially approved procedures for the care and use of laboratory animals, and all protocols met the requirements of the local ethical committee of Bar Ilan University, Israel. The mice were fed *ad libitum* and allowed free access to drinking water. The temperature and relative humidity were kept constant at 20-24°C and 60%, respectively. The health status of the animals used in the experiments was examined on arrival. Only animals in good health were acclimatized to laboratory conditions for 7 days prior to each study initiation.

Animals were monitored and observed for the total duration of the experiments. Viability checks for mortality and morbidity were performed at least once daily whereas detailed clinical signs were performed twice a week. Clinical signs observations included changes in skin, fur, eyes, mucous membranes, occurrence of secretions and excretions (e.g., diarrhea) and autonomic activity (e.g., lacrimation, salivation, piloerection, pupil size, unusual respiratory pattern). Changes in gait, posture and response to handling, as well as the presence of bizarre behavior, tremors, convulsions, sleep and coma were also observed and recorded. All animals were humanely euthanized at the end of the experiments.

### Sarah Nanotechnology Treatment

SaNPs at concentrations between 1.6-2.1 mg IO/mL were supplied as a dispersion in water for injection (WFI) and administered to the mice via an IV bolus injection to the lateral tail vein. The volume of the injection was decided after weighing each mouse, 10 µl NP per 1 gr of mouse, which is the maximal feasible dose in mice. All test materials were injected using an insulin syringe and a 27G needle.

Alternating current magnetic field (AMF) application was conducted using an electromagnetic induction system (EIS) comprising of 3 main components: an electromagnetic coil, an AMF generator, and a chiller. The EIS generated a magnetic field at an amplitude range of 8-33 kA/m. A polypropylene perforated housing 50-mL tube was used to hold the mouse, without anesthesia, while in the system’s inductor coil. This tube provided shield so that the mouse could not come in direct contact with the coil and kept all mice positioned in the same direction. Each mouse was inserted into the housing tube, which was then placed in the coil. The coil was powered by the generator and cooled by running water kept at 20°C by the chiller (Tek-Temp Instruments, PA, USA). AMF application commenced 2-8hrs post SaNP administration. Upon activation of the AMF at 290 ± 10% kHz, the mice were irradiated for a total of 30 min.

### Biodistribution

The short-term biodistribution of SaNPs in the target organ (lungs) and blood over time, was assessed at 3 different timepoints, 2, 4, and 8hrs, following a single IV bolus injection of SaNPs (2.1 mg IO/mL) to the tail vein of 12 BALB/c female mice bearing 4T1 breast cancer metastatic tumors to determine the optimal time for AMF exposure. Five mice were weighed and assigned to each group. At termination time the lungs from each mouse were excised, weighed and stored frozen separately. Blood was collected in EDTA-tubes (Greiner Bio-One GmbH, Austria), the volumes were measured and recorded, and samples were stored at 4°C until analysis.

The IO content in the lungs and blood was determined in the specimens by superconducting quantum interference device (SQUID) analysis that measures the magnetic properties of nanoparticles and enables detection in organic samples with high sensitivity (14). This method was used in our initial studies. Magnetization measurements were collected using a Quantum Design MPMS-XL5 SQUID magnetometer at 300 K (Bar-Ilan University, Israel). Before the analysis, the lungs were homogenized using a BeadBug microtube homogenizer (Benchmark Scientific, NJ, USA). The homogenates (100 μl) were transferred into polycarbonate capsules and subjected to lyophilization. The amount of SaNPs in the lungs and blood was determined using a calibration curve built based on the quantification of magnetic moment values as a function of concentration of known SaNP dilutions. The corresponding SaNP concentration within a sample was calculated by normalizing the NP amount to the tissue (whole organ) weight or blood volume. SQUID measurements were performed for each individual mouse to demonstrate reproducibility among animals.

The long-term biodistribution and excretion of SaNP in vital organs and blood was evaluated over time. Twenty healthy BALB/c female and male mice were weighed and treated with Sarah Nanotechnology comprising of a single SaNP IV injection (1.7 mg IO/mL), followed by 30 min of AMF application (16.7 kA/m) at 8hrs post injection to examine the SaNPs’ fate within the context of the whole approach (e.g., full treatment). Each group was assigned 10 animals, 5 male and 5 female mice. The animals were sacrificed at 4 and 13 weeks post a single treatment. All animals were subjected to blood sampling and organ harvesting at their respective scheduled termination. A volume of at least 300 μl whole blood, collected into lithium heparin-coated tubes (Greiner Bio-One GmbH, Austria) was individually obtained from the mice. Blood samples were refrigerated (2-8°C) until all organs were processed following termination.

Organ collection included the following: brain, spleen, kidneys, lungs, heart, liver, mandibular and medial iliac lymph nodes, all weighed wet as soon as possible following their dissection and individually fixed in pre-labeled tubes containing 10% neutral buffered formalin (NBF). The fixed organs were cut into several sections using disposable equipment, to avoid cross-contamination. The processing of the organs was done separately for each follow up period. Each section was individually placed in a pre-weighed tube, containing the organ section, and weighed again. The samples were analyzed for the quantitation of IO content by particle electron paramagnetic resonance (pEPR), using a pEPR analyzer. The pEPR technique has been validated, is based on a low-field and low-frequency electron paramagnetic resonance, measures the magnetization of super paramagnetic iron oxide nanoparticles (SPIONs), and enables their quantitation (15). The bioanalysis of samples collected for long-term biodistribution evaluation was performed by Pepric (Leuven, Belgium). To generate a calibration curve, known SaNP dilutions were prepared and measured by pEPR to establish the limit of detection (LoD) for each set of samples. Then, all samples were measured and a magnetic signal per volume (μL) was generated for each tube. The signal was then normalized in accordance to the tissue weight or blood volume in each tube and calculated for each organ to obtain the SaNP volume in the whole organ. SaNP percentages in blood and each organ were calculated.

### Toxicity

The potential toxic effects of SaNPs were assessed following a single IV bolus injection (2.1 mg IO/mL) to BALB/c healthy mice followed by AMF application (33 kA/m), to examine the toxicity of the full treatment. Thirty mice were subjected to observation and terminated at 3 different timepoints, 3, 14, and 30 days after treatment, control mice remained untreated. Five mice were weighed and assigned to each group.

A separate repeated dose chronic toxicity study was conducted in female BALB/c mice for a longer follow up period of 25 weeks. This study included 30 mice that received either vehicle control (5% glucose) or 3 IV injections of SaNPs (1.6 mg IO/mL) at an interval of one month combined with AMF application (33 kA/m) after each dosing session. Fifteen mice were weighed and assigned to each group. Five mice from the treated group were subjected to IO content evaluation by the pEPR method as described above.

Measurements post sacrifice included blood analyses (hematology, chemistry), necropsy, gross pathology, and histopathology of vital organs. Blood analyses were conducted by the American Medical Laboratories (AML), Ltd, Herzelia, Israel. Blood was collected from the orbital sinus and spun down to separate serum, the blood and serum were stored at 4°C until analysis. Indicators of liver and kidney functions such as alanine aminotransferase (ALT), aspartate transaminase (AST), total bilirubin, albumin, gamma-glutamyl transferase, alkaline phosphatase, and urea were monitored. White blood cells (WBC), red blood cells (RBC), hemoglobin, mean cell hemoglobin (MCH), mean cell volume (MCV), polymorphonuclear cells, lymphocytes, and platelets were also determined. The following tissues were collected at necropsy: heart, lung, liver, kidneys, spleen, long bone, and brain and kept in 10% NBF until sectioning.

### Efficacy

The efficacy of Sarah Nanotechnology treatment was evaluated following 3 treatment cycles, in the murine 4T1 mCherry breast cancer metastatic model in BALB/c female mice that were weighed and randomly divided. Each group, control and treatment, was assigned 5 mice. The treatment cycles (SaNP injection, 1.8 mg IO/mL, followed by 30 min of continuous AMF application, 33 kA/m, at 8hrs post injection) started at day 10 post cell inoculation, and were applied within 2-days intervals between each cycle. The primary endpoint of the study was to record the number and size of metastases in the lungs. This was achieved by visual counting, determination of the metastases’ fluorescence intensity, and histopathology efficacy evaluation. At the end of the study, the animals were sacrificed, the lungs were excised and the number of metastases was visually counted followed by fluorescence *ex-vivo* imaging using the CRi Maestro™ multispectral imaging system (Cambridge Research & Instrumentation, Inc.). The number of tumors/nodules in the lungs was reported, expressed either as single, or multiple nodules. A two-dimensional morphometric measurement and average area quantitation (mm^2^) was done on the largest nodule (i.e., tumor) present in the lung sections based on the recommendations of the Fleischner Society for measuring pulmonary nodules at CT (16) which recommend to measure only the greatest dimension of the largest solid component. The morphometric evaluation was performed using the calibrated Augmentiqs system (https://www.augmentiqs.com/) as described (17).

### Survival

Ten BALB/c mice bearing mCherry breast cancer metastatic tumors were randomly assigned 5 to a group, weighed, and treated with 5 cycles of Sarah Nanotechnology, to mimic a chronic treatment setting, starting at day 13 post cell inoculation, each including a single IV bolus SaNP dose (1.8 mg IO/mL) to the tail vein, followed by 30 min of AMF application (33 kA/m) at 8hrs post injection. The experiment concluded when the last mouse died. The date of each animal’s death was recorded. The primary endpoint of the study was the survival of the mice; treated *vs*. untreated control.

### Histopathology Analysis

Histological slides were prepared by Patho-Lab Diagnostics Ltd., Ness Ziona, Israel. Tissues harvested for microscopic examination were fixed in 10% NBF for at least 24hrs. Lungs were inflated by formalin to their normal volume. Tissues were trimmed, according to the registry of industrial toxicology animal-data (RITA) standards (18), and dehydrated through a graded series of alcohols and cleared in xylene. Tissues were embedded in paraffin, sectioned at 5-6 μm, and stained with hematoxylin and eosin (H&E).

For the detection of IO nanoparticles, selected tissues (i.e., liver, lungs) were stained with Prussian blue (19). Stained slides were examined with an Olympus BX-51 microscope (Olympus, Melville, NY, USA). The evaluation was done in a blinded manner, i.e., without knowing the treatment in each group. Any histopathological findings were recorded, described and scored by a Board- certified study Pathologist, using semi-quantitative grading of five grades (0–4), taking into consideration the severity of the changes (0 = No Lesion, 1 = Minimal Change, 2 = Mild Change, 3 = Moderate Change, 4 = Marked Change) (20).

### Statistical Analysis

All data are expressed as the mean ± S.D. The statistical significance of differences between groups was analyzed by Student’s *t* test and a *p*-value of < 0.05 was considered to be statistically significant. Comparison of results among groups was carried out by one-way analysis of variance (ANOVA). Kaplan-Meier statistical analyses were utilized to compare survival.

## Results

### Characterization of Nanoparticles

**Figure 1:**
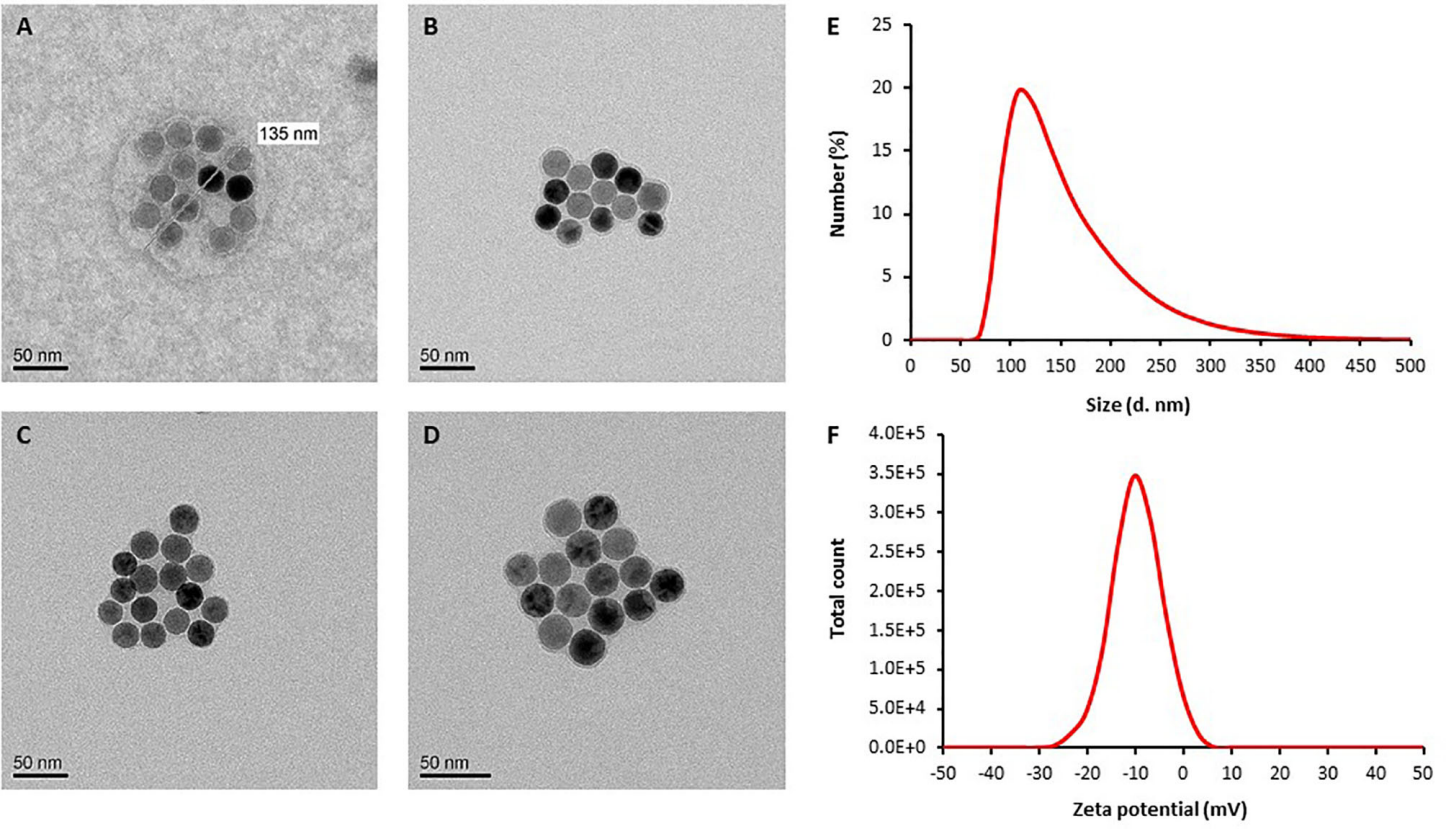
Representative TEM images of SaNPs. **(A)** SaNP with spherical shape. **(B–D)** SaNPs with amorphous (non-spherical) shape. Images were obtained using a Tecnai G2Spirit Twin T-12 electron microscope with an accelerating voltage of 120 kV. **(E)** Size (diameter) distribution of SaNPs (d. nm) determined by DLS. **(F)** Distribution of zeta potential values determined by DLS (mV).

TEM imaging demonstrated that SaNPs, containing several encapsulated 25 nm IO nanoparticles, exhibit a monodisperse state and amorphous or spherical shapes. The variability in shape arises from the flexible nature of the nanoparticles. Images of SaNPs with diverse shapes captured by TEM are shown in **Figures 1A–D**. TEM and DLS measurements showed an average size of 135 ± 25 nm and zeta potential values of (5)-(-30) mV, respectively (**Figures 1E, F**). The SaNPs’ surface electric charge was measured to have a negative value of -(9.7) ± 1.2 mV at neutral pH as shown in **Figure 1F**.

### Accumulation of SaNPs in Lungs Bearing Metastases

**Figure 2:**
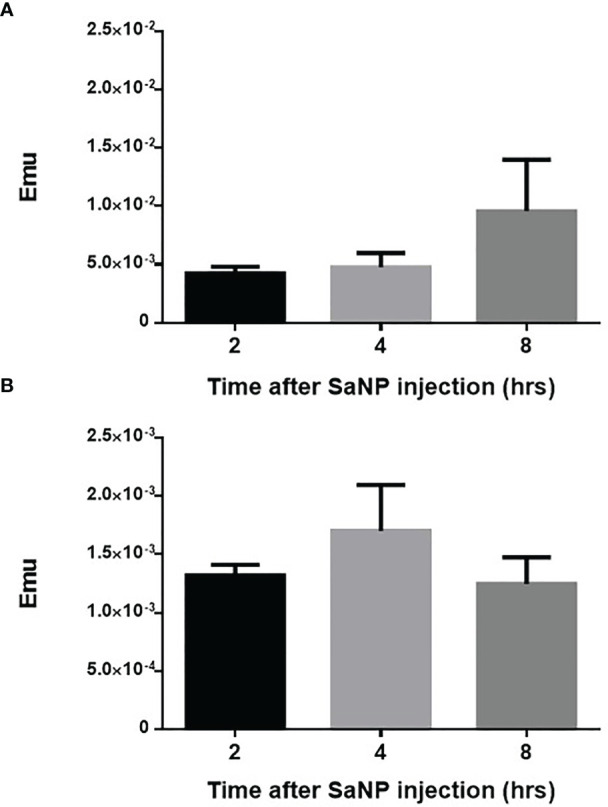
Biodistribution of SaNPs in the lung metastases and blood. **(A)** SaNP accumulation in the lungs of BALB/c mice-bearing 4T1 metastases at 2, 4, and 8hrs post injection. **(B)** SaNP accumulation in the blood at 2, 4, and 8hrs post injection. Samples were analyzed by SQUID for the quantitation of IO content. Results are expressed as mean ± S.D. electromagnetic units (EMU).

The purpose of the short-term biodistribution study was to examine the accumulation of SaNPs in the target organ, thereby establishing the optimal time for AMF application after SaNP administration. We assumed that due to the EPR effect, the SaNPs were preferentially retained in the lung metastases. The results indicated that the amount of SaNP that reaches the lungs was 2-fold higher and the highest at 8hrs post injection (**Figure 2A**) whereas the concentration in the blood was the highest at 4hrs after SaNP administration and decreased thereafter (**Figure 2B**). Thus, the accumulation of SaNPs in the lungs increased over time and was the highest when the concentration of SaNP in the blood decreased.

### SaNPs Accumulate in the Liver and Spleen

**Figure 3:**
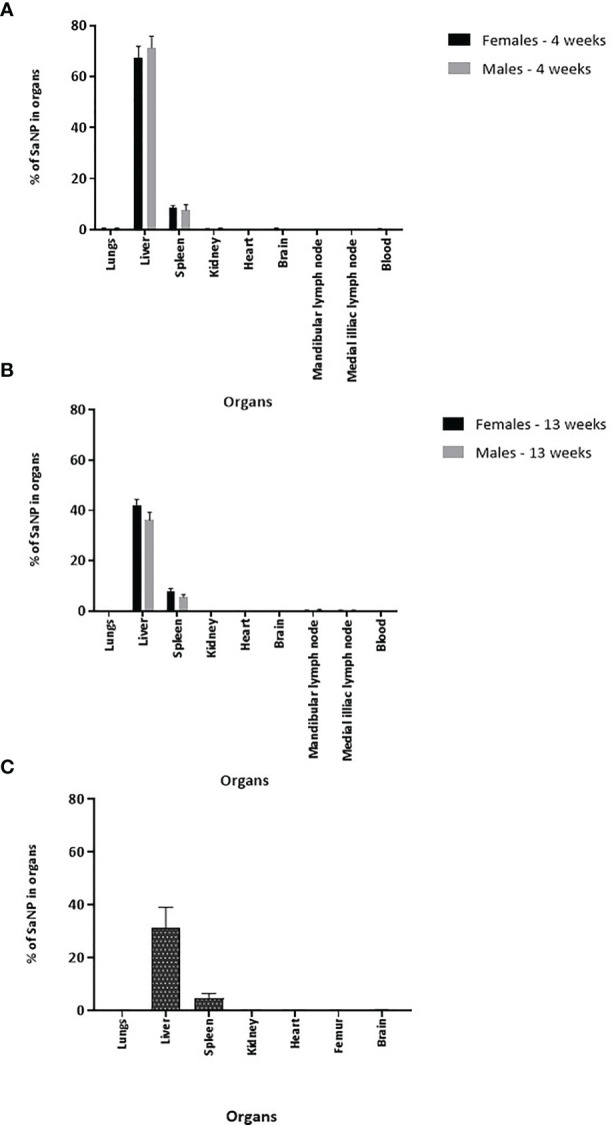
Long-term tissue biodistribution of SaNPs. **(A)** Residual SaNP in the organs and blood of BALB/c mice at 4 weeks after a single treatment. **(B)** Residual SaNP at 13 weeks after a single treatment. **(C)** Residual SaNP at 25 weeks after 3 repeated treatments. Samples were analyzed by pEPR for the quantitation of IO content. Results are expressed as the percentage of SaNP in the various organs and blood normalized per mg/tissue.

The biodistribution analysis revealed that the percentage of total residual SaNP left in the sampled organs and blood was 77.1% in female and 79.8% in male mice after 4 weeks, and 50.2% in female and 42.2% in male mice after 13 weeks. An average of 78.4% and 46.2% was found after 4 and 13 weeks, implying that 21.5% and 53.8%, were cleared from the animals’ bodies after these time periods, respectively (**Figure 3**). The difference between the two timepoints was statistically significant (*p*-value<0.0001). The results demonstrated that SaNPs were primarily accumulated in the MPS, with the liver having the highest SaNP accumulation per organ weight. This is to be attributed to the large number of resident tissue macrophages (i.e., Kupffer cells) in the liver, followed by the spleen (21).

The percentage of total residual SaNP left in the liver after 4 weeks was 67.5 ± 4.4% in female and 71.2 ± 4.6% in male mice whereas the percentage left in the liver after 13 weeks was 41.8 ± 2.5% in female and 36.1 ± 3.2% in male mice. There was a time-dependent decrease in the total residual SaNP left in the liver, the main accumulation site, of about 38% in female and 50% in male mice between 4 and 13 weeks. The percentage of total residual SaNP left in the spleen after 4 weeks was 8.5 ± 0.9% in female and 7.6 ± 2.2% in male mice. After 13 weeks, the percentage left in the spleen was 7.8 ± 1.1% in female and 5.5 ± 1% in male mice. The spleen, the secondary accumulation site, had a time-dependent decrease of 0.7% and 2.1% in female and male mice, respectively.

Of note, the percentage of total residual SaNP left in the lungs after 4 weeks was 0.3 ± 0.3% in female and 0.4 ± 0.2% in male mice and after 13 weeks, the percentages were 0.1 ± 0.05% in female and 0.07 ± 0.05% in male. No residual SaNPs were found in the kidneys, heart, brain, lymph nodes, and blood. The individual findings for the selected organs and blood at the two timepoints, 4 and 13 weeks, are presented in **Figures 3A, B**, and expressed as mean percentage of SaNP (% SaNP) ± standard error of mean (SEM) in the corresponding tissue. The results are representative of two different experiments.

In a separate study, examining the SaNP biodistribution in organs and blood after 25 weeks in mice that received 3 repeated SaNP doses, to mimic a chronic treatment setting, it was found that 36.3% of the original 3 SaNP doses remained in the mouse organs after the initial SaNP injection. Thus, 63.7% of the injected SaNPs were cleared from the mice body after 25 weeks. The highest percentage of SaNPs accumulated in the liver (31.4 ± 7.6%) and spleen (4.5 ± 1.90%), consistent with the previous findings. The average percentage of SaNPs that accumulated in the lungs of female mice was relatively low, (0.07 ± 0.07%) whereas the percentage of SaNPs found in the kidneys, heart, and brain was minor and comparable (**Figure 3C**).

### SaNPs Are Not Toxic Upon Systemic Administration

**Figure 4:**
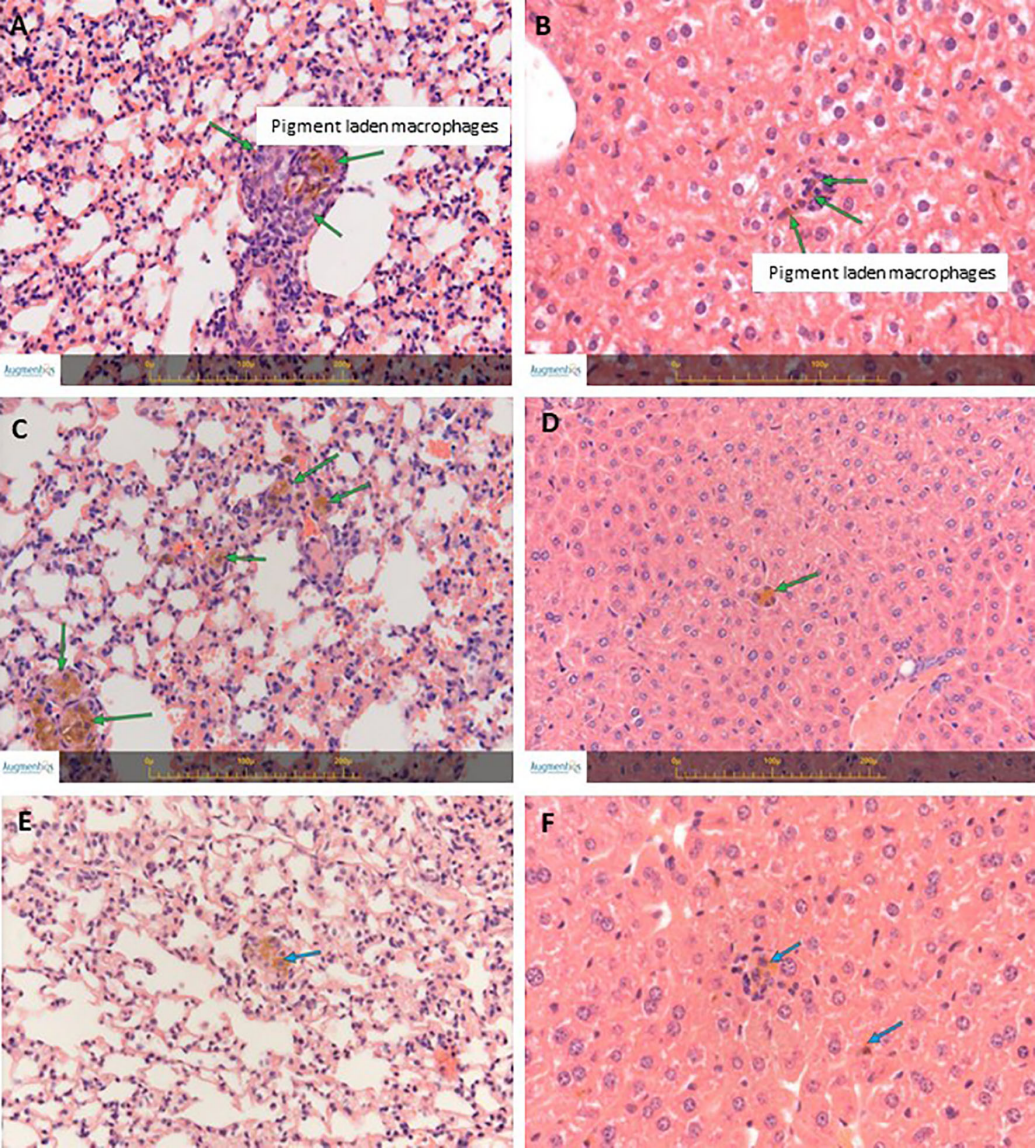
H&E staining of lungs and livers from naïve BALB/c treated mice. **(A)** Lungs’ section from a mouse at 3 days post treatment. **(B)** Liver section from a mouse at 3 days post treatment. **(C)** Lungs’ section from a mouse at 14 days post treatment. **(D)** Liver section from a mouse at 14 days post treatment. **(E)** Lungs’ section from a mouse at 30 days post treatment. **(F)** Liver section from a mouse at 30 days post treatment. Arrows indicate small collections of pigment laden macrophages in the liver (Kupffer cells) and in the lungs, associated with minimal mononuclear cell infiltration. Images were captured using the Augmentiqs system software (14). Results are representative of 3 different experiments.

Next, we examined the potential toxic effects of Sarah Nanotechnology treatment following a single dose IV bolus injection of SaNPs to BALB/c naïve mice and AMF application at 8hrs post injection. Treated and untreated (control) mice were sacrificed at 3, 14, and 30 days after treatment. All animals were subjected to a full detailed necropsy and gross pathological examination following the respective scheduled termination. No mortality occurred in any of the animals throughout all observation periods. The mice well tolerated the SaNP dose with no clinical signs of toxicity after the injections. No significant changes in body weight were observed nor gross pathological findings were evident in any of the treated animals at their scheduled necropsy. Histopathological analysis of organs, indicated that no treatment-related changes were found in the organs examined (kidneys, spleen, heart, brain). Minimal pigment laden macrophages (i.e., Kupffer cells), which were associated with minimal inflammatory (mononuclear) cell infiltration were noted in the liver and lungs of the treated animals at all timepoints. As the changes in these organs were of minimal degree, they were not considered as adverse (22, 23). Representative images of the liver and lungs are shown in **Figure 4**.

Blood analyses showed minor changes in the blood cell counts of the treatment *vs*. control group that were not considered of clinical relevance and were of no concern. Changes were detected in liver enzymes, mainly in ALT and AST at the 3 days timepoint only, which were increased in the treatment compared to the control group. The complete blood analysis biochemical profile of the animals at the day 3 timepoint is shown in **Table 1**. The results indicate that the levels of creatinine, calcium, phosphate, glucose, urea, cholesterol, total protein, albumin, globulin, alkaline phosphatase, and electrolytes in blood (Na, K, and Chloride) were similar in the control and treatment groups. AST values at the 3 days timepoint were 5-fold higher in the treated group compared to the control mice (*p-*value>0.05) whereas ALT values at the same timepoint were 3.7-fold higher in the treated group compared to the control (*p*-value>0.05). However, while evaluating the individual parameters, the increase was due to unusually high levels of these parameters in two of the treated animals. In the rest of the treated animals, the range of the values was comparable to those seen in the control group. In one of the treated animals, the values of AST were even lower than that seen in the control group.

**Table 1:** Blood analysis biochemical profile of mice at the day 3 timepoint.

Group	Animal No.	Creatinine	Calc	Phos	Glucose	Urea	Chol	T Protein	Alb	Glob	T Bilirubin	Alk Phos	AST	ALT	Na	K	Chlor
		mg/dl	mg/dl	mg/dl	mg/dl	mg/dl	mg/dl	g/dl	g/dl	g/dl	mg/dl	IU/L	IU/L	IU/L	mmol/L	mmol/L	mmol/L
**Control**	**1**	NA	9.05	10.9	196	62.5	72	4.86	3.4	1.46	<0.146	159	458	170	153	6.1	115
	**2**	0.16	9.2	4.8	269	39.8	71	4.31	3.1	1.21	0.05	157	136	47	149	3.6	112
	**3**	0.18	8.72	12.5	242	40.1	67	4.3	2.8	1.5	0.04	154	238	47	151	5.7	115
	**4**	0.12	8.75	5.6	231	30.9	66	4.37	3.2	1.17	0.08	197	127	64	151	3.2	115
	**5**	-	-	-	-	-	-	-	-	-	-	-	-	-	-	-	-
	**Average**	**0.15**	**8.93**	**8.45**	**234.50**	**43.33**	**69.00**	**4.46**	**3.13**	**1.34**	**0.06**	**166.75**	**239.75**	**82.00**	**151.00**	**4.65**	**114.25**
	**STDEV**	**0.03**	**0.23**	**3.82**	**30.23**	**13.48**	**2.94**	**0.27**	**0.25**	**0.17**	**0.02**	**20.27**	**153.96**	**59.21**	**1.63**	**1.46**	**1.50**
**Treatment**	**6**	0.09	6.67	7	260	46.9	72	3.99	2.9	1.09	0.06	186	363	223	150	4.4	111
	**7**	0.13	7.47	6.9	280	48.5	69	4.01	3	1.01	0.02	152	218	120	150	4.6	114
	**8**	0.26	5.55	13.6	214	50.8	57	3.54	2.4	1.14	0.03	113	2613	714	144	11.4	111
	**9**	0.26	7.86	6.7	338	44.8	81	4.34	3.3	1.04	0.05	116	117	66	148	3.8	111
	**10**	0.31	7.03	17.2	200	53.1	65	4.21	2.9	1.31	0.01	130	2672	422	149	12.4	110
	**Average**	**0.21**	**6.92**	**10.28**	**258.40**	**48.82**	**68.80**	**4.02**	**2.90**	**1.12**	**0.03**	**139.40**	**1196.60**	**309.00**	**148.20**	**7.32**	**111.40**
	**STDEV**	**0.09**	**0.89**	**4.85**	**55.20**	**3.25**	**8.84**	**0.30**	**0.32**	**0.12**	**0.02**	**30.26**	**1322.98**	**264.08**	**2.49**	**4.21**	**1.52**

NA, Not available; T, Total.

No changes were detected later on, at the 14 and 30 days timepoints. The changes in ALT and AST levels at the 3 days timepoint were thought to reflect an incidental biological variation, were not related to any hepatocytic damage, and are in correlation with the pigment accumulation observed in the liver by the histopathology evaluation (**Figure 4**).

**Figure 5:**
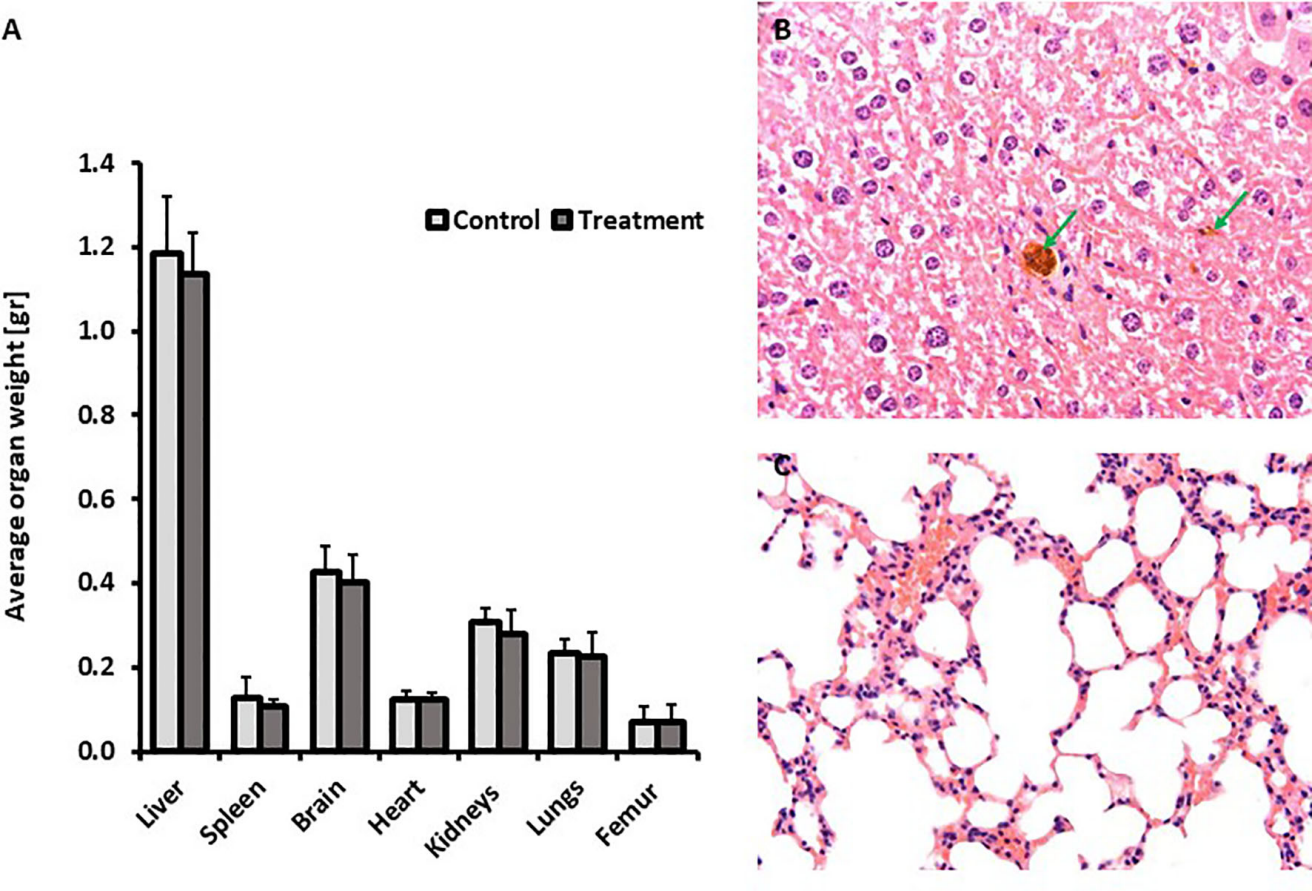
Repeated dose chronic toxicity study. **(A)** Average organ weight after a follow up period of 25 weeks. **(B)** Representative liver section (H&E staining) from a treated mouse at 25 weeks post treatment. Green arrows indicate pigment laden macrophages in the liver (Kupffer cells) associated with minimal mononuclear cell infiltration. **(C)** Representative lungs’ section (H&E staining) from a treated mouse at 25 weeks post treatment. No changes were observed. Images were captured using the Augmentiqs system software (14).

The results of the chronic toxicity study highlighted that 3 repeated IV doses of the SaNP followed by AMF application did not cause any adverse effects on the general health status and body weight of all treated mice compared to the controls. All animals exhibited normal body weight gain at the end of the follow up period. The average organ weights (**Figure 5A**) of the treatmet group were comparable to those of the control group. Statistically significant changes were not observed between the two groups. A single case of mortality occurred on day 84 in the control group, and was therefore not considered related to treatment. No noticeable clinical signs in reaction to treatment were evident throughout the entire observation period and there were no significant differences observed between the control and treated animal groups in clinical pathology parameters. Blood hematology analyses that included blood cell counts (RBC, WBC, lymphocytes, monocytes, eosinophils, basophils, platelets, neutrophils) and hematological values (hemoglobin, hematocrit, MCV, MCH, MCHC) at 25 weeks post treatment were all within normal ranges. Chemistry analysis indicated that values of creatinine, phosphate, urea, cholesterol, total protein, albumin, globulin, total bilirubin, sodium, and chloride at 25 weeks were within normal ranges as well. The levels of liver enzymes, ALT and AST were higher in animals of the control group compared to the treatment mice, probably due to two outliers in this group. These deviations were considered to be unrelated to SaNP administration and AMF application. No adverse histopathological effects related to treatment were observed in the organs at the tested dose level. The average values of the complete blood analysis results of the animals, hematology and chemistry, are shown in **Tables 2**, **3**, respectively. Minor treatment related changes appeared only in the liver, consisting of minimal brownish pigment accumulation within the Kupffer cells (macrophages), and minimal mononuclear cell infiltration, which was not associated with any necrotic, fibrotic or hyperplastic lesions, similar to the aforementioned findings in the shorter observation periods. Moreover, the changes in the liver did not show any time-related increased severity and they consistently remained minimal with no progressive changes, and therefore not considered as adverse (**Figures 5B, C**). Notably, at this timepoint, no changes involving pigment accumulation nor mononuclear cell infiltration were observed in the lungs, suggesting a time-related recovery of this organ.

**Table 2:** Average values of hematology blood analysis results of mice at 25 weeks post treatment.

Group		WBC	RBC	HGB	Hematocrit	MCV	MCH	MCHC	Neut	Lymph	Mono	Eos	Baso	Platel
		10*3/µl	10*6/µl	g/dl	%	fL	pg	g/dl	%	%	%	%	%	10*3/µl
**Control**	**Average**	5.36	9.04	13.62	40.95	45.53	15.10	33.19	15.46	81.62	2.31	0.62	0.00	657.85
	**STDEV**	2.31	1.98	2.87	8.43	1.89	0.52	1.05	7.15	6.99	3.35	0.96	0.00	403.88
**Treatment**	**Average**	5.79	9.52	14.09	42.62	44.84	14.78	33.03	16.00	81.08	1.69	1.23	0.72	788.08
	**STDEV**	3.66	1.63	2.42	7.08	1.38	0.17	1.17	8.21	8.31	1.97	1.74	2.61	262.53

**Table 3:** Average values of chemistry blood analysis results of mice at 25 weeks post treatment.

Group		Creatinine	Phos	Glucose	Urea	Chol	T Protein	Alb	Glob	T Bilirubin	AST	ALT	Na	Chlor
		mg/dl	mg/dl	mg/dl	mg/dl	mg/dl	g/dl	g/dl	g/dl	mg/dl	IU/L	IU/L	mmol/L	mmol/L
**Control**	**Average**	0.22	7.09	263.18	46.40	69.36	4.20	2.86	1.33	0.04	702.36	385.45	148.36	113.64
	**STDEV**	0.04	2.67	45.97	8.64	18.35	1.00	0.74	0.41	0.02	1220.62	718.65	8.52	4.76
**Treatment**	**Average**	0.25	7.30	254.67	44.83	74.08	4.36	3.01	1.35	0.06	282.75	141.33	143.50	113.42
	**STDEV**	0.06	1.72	69.14	7.40	16.64	0.81	0.49	0.35	0.03	424.55	220.66	19.84	2.71

T, Total.

### SaNPs Show Therapeutic Outcome

**Figure 6:**
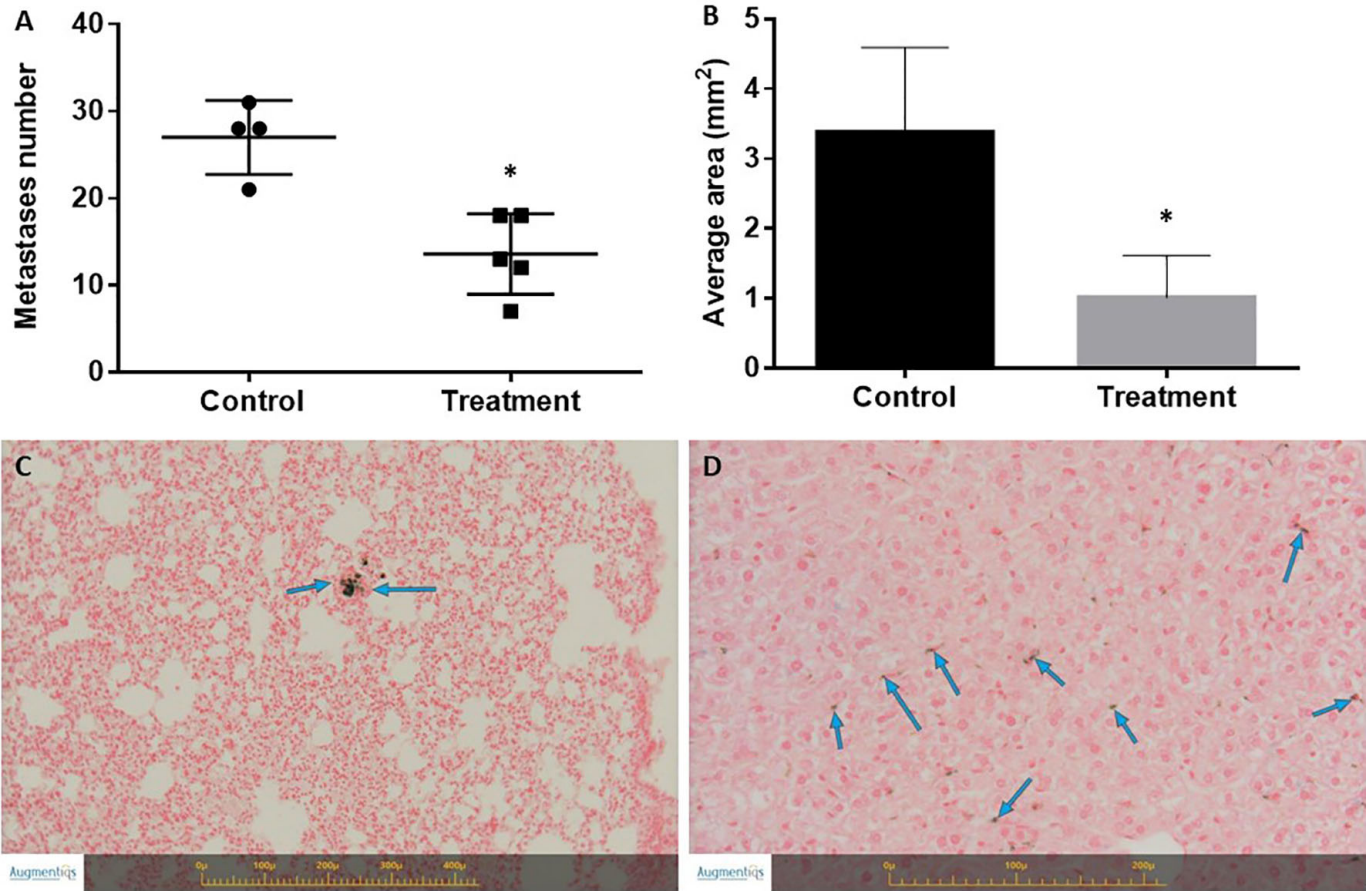
Effect of treatment on the number of lung metastases and tumor size in the 4T1 breast cancer model. **(A)** Number of lung metastases. Mice were sacrificed on day 17 following cancer model induction. The lungs were excised and the number of metastases was counted. *Statistically significant difference. **(B)** Tumor size determined by histopathology and 2-D morphometric analysis expressed as average tumor area (mm^2^). *Statistically significant difference. **(C)** Representative section of Prussian blue staining of the lungs of treated mice. Blue arrows indicate Prussian blue positive pigment laden macrophages. No inflammation was associated with the presence of pigment. **(D)** Representative section of Prussian blue staining of the liver of treated mice. Blue arrows indicate Prussian blue positive pigment laden macrophages (Kupffer cells). No inflammation was associated with the presence of pigment. Images were captured using the Augmentiqs system software (14).

The efficacy of Sarah Nanotechnology treatment was investigated in the 4T1 mCherry breast cancer metastatic model. Control animals were injected with 5% glucose (vehicle). A significant reduction in the number of metastases was identified in BALB/c mice bearing 4T1 metastatic tumors following 3 treatment cycles of Sarah Nanotechnology within 2 days intervals (**Figure 6A**). The average number of metastases was 22.5 ± 10.7 in the control and 13.6 ± 4.6 in the treatment group. There was a 2-fold decrease in the treatment *vs*. control mice (*p*-value<0.05). The two-dimensional morphometric measurement on the largest nodule (i.e., tumor) present in the lung sections revealed significant differences in the mean tumor area of the treated mice. The calculated relative average area of the measured largest metastatic nodule in this group was 3.3795 ± 1.219 mm^2^ in the control compared to 1.0116 ± 0.602 mm^2^ in the treatment mice (**Figure 6B**), showing a ~3-fold decrease (*p*-value<0.05). Of note, only in the lungs of treated animals, minimal accumulation of pigment laden macrophages was noted as demonstrated by positive Prussian blue staining which indicates IO deposition (**Figure 6C**). Furthermore, only in the liver of treated animals, minimal accumulation of pigment laden macrophages (i.e., Kupffer cells) was observed (**Figure 6C**). The accumulation of the pigment was not associated with any necrosis and/or fibrosis in the lungs or liver. In the spleen, the same relative amount of Prussian blue pigmented macrophages was found in the red pulp, in the control and treated animals (results not shown), reflecting normally present hemosiderin pigmentation. In particular, the spleen was examined for any potential morphological changes according to the recent Enhanced Immunotoxicology standards used for the pathology evaluation of the immune system, and no treatment related histopathological nor abnormal changes were noted (24). Slight changes, within normal reference range, were detected in the levels of RBC, lymphocytes, neutrophils, urea, cholesterol and AST by blood and chemistry analyses. The average values of the complete blood analysis results of the animals, hematology and chemistry, are shown in **Tables 4**, **5**, respectively. None of these changes was considered of concern. Moreover, no adverse reaction, inflammation and/or necrosis were noted in any of the organs of the treated animals.

**Table 4:** Average values of hematology blood analysis results of control *vs*. treated mice following 3 treatment cycles with Sarah Nanotechnology system.

Group		WBC	RBC	HGB	Hematocrit	MCV	MCH	MCHC	Neut	Lymph	Platel
		10*3/µl	10*6/µl	g/dl	%	fL	pg	g/dl	%	%	10*3/µl
**Control**	**Average**	6.27	6.33	10.86	32.91	42.29	14.16	27.00	35.44	47.44	212.03
	**STDEV**	3.43	4.15	6.78	21.40	22.86	8.36	15.56	23.07	28.09	190.95
**Treatment**	**Average**	6.94	10.00	14.78	51.56	51.56	14.78	28.64	29.40	70.60	201.49
	**STDEV**	1.86	0.44	0.62	1.87	1.44	0.11	0.68	7.20	7.20	14.90

**Table 5:** Average values of chemistry blood analysis results of control *vs*. treated mice following 3 treatment cycles with Sarah Nanotechnology system.

Group		Calc	Phos	Glucose	Urea	Chol	T Protein	Alb	Glob	T Bilirubin	Alk Phos	AST	ALT	Na	K	Chlor
		mg/dl	mg/dl	mg/dl	mg/dl	mg/dl	g/dl	g/dl	g/dl	mg/dl	IU/L	IU/L	IU/L	mmol/L	mmol/L	mmol/L
**Control**	**Average**	7.79	7.19	65.58	72.48	68.55	4.10	2.84	1.31	0.05	220.76	912.93	433.60	122.14	7.99	82.30
	**STDEV**	3.98	3.78	26.32	49.69	34.30	2.22	1.43	0.69	0.02	88.93	477.09	312.47	64.18	4.03	41.04
**Treatment**	**Average**	10.97	9.40	82.00	45.08	106.67	5.37	3.53	1.73	0.04	278.00	1282.67	534.25	151.75	8.63	102.50
	**STDEV**	1.11	1.56	20.88	2.00	16.77	0.16	0.30	0.10	0.04	68.79	706.64	290.00	7.68	1.56	10.50

T, Total.

**Figure 7:**
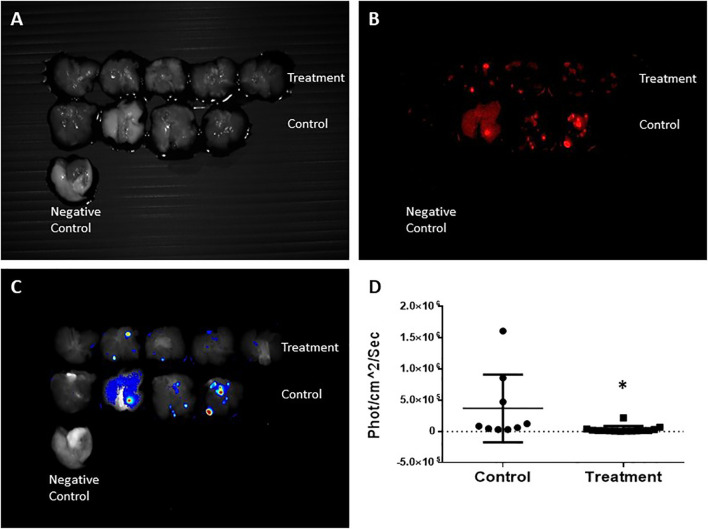
CRi Maestro fluorescent *ex-vivo* imaging of the lungs. **(A)** Lungs – grayscale. **(B)** Fluorescent imaging of mCherry metastases. **(C)** Heat map of viable metastases. **(D)** Quantitation of total fluorescent signal in the lungs per group, expressed in x10^6^phot/cm^2^/sec. Each dot represents the fluorescence intensity of individual metastases. Normal lungs without mCherry expression were used as a negative control. Each pair of lungs may contain several metastases that express different fluorescence intensities. Results are representative of 3 different experiments. *Statistically significant difference.

*Ex-vivo* imaging of the lungs was conducted using the CRi Maestro™ multispectral imaging system (**Figure 7**). Lung metastases of 4T1 mCherry breast cancer cells were clearly visible as demonstrated by the bright red signal shown in **Figure 7B**. A heat map of the viable metastases is provided in **Figure 7C** showing that the lungs of the control mice were more viable than those of the treated mice as indicated by the colors in the map. The total fluorescent signal (x10^6^ phot/cm^2^/sec) of the lungs in the treated mice, indicative of viability, was significantly lower than that of the control mice. The distribution of the fluorescence in the lungs is shown in **Figure 7D** (*p-*value 0.0241).

**Figure 8:**
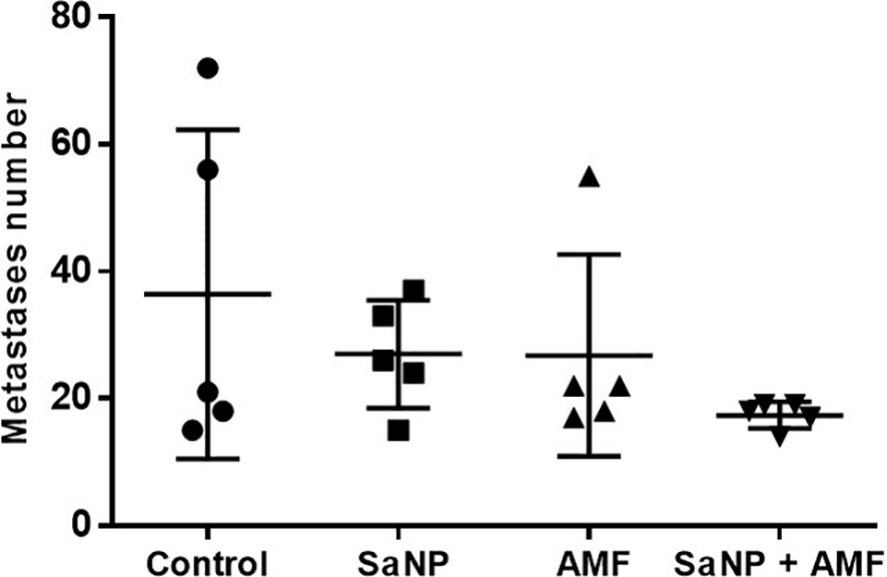
Effect of treatment on the number of lung metastases in the 4T1 breast cancer model. Treatment cycles started at day 14 after cell inoculation. Mice were sacrificed on day 21 post treatment. The lungs were excised and the number of metastases was counted.

Of note, a previous initial study aimed to establish the primary mode of action of the Sarah Nanotechnology system, examined the effects of SaNP alone (1.8 mg IO/mL), AMF alone (33 kA/m, 290 ± 10% kHz), and SaNP + AMF application at 8hrs post injection on the number of lung metastases, following 3 treatment cycles within 2 days intervals. Five mice were assigned to each group. The results presented in **Figure 8**, demonstrated a reduction in both the average number of metastases and the corresponding standard deviation of the full treatment (SaNP + AMF) compared to the control group.

A two-way ANOVA was conducted showing no statistically significant differences between the groups, probably due to the small number of animals (n=5/group). However, despite the lack of statistical significance, these results suggest that only the combination of SaNP injection and AMF application (full treatment) leads to a therapeutic effect involving a reduction in the number of metastatic nodules in the lungs of BALB/c mice.

### SaNPs Improve the Survival of 4T1 Tumor Bearing Mice

To investigate the effects of Sarah Nanotechnology treatment on animal survival in the 4T1 lung metastasis model, the mice were treated 13 days after cancer cell inoculation. Control animals were injected with 5% glucose (vehicle). The results established that following 5 treatment cycles of Sarah Nanotechnology treatment, the survival of treatment mice was significantly improved compared to that of the control.

**Figure 9:**
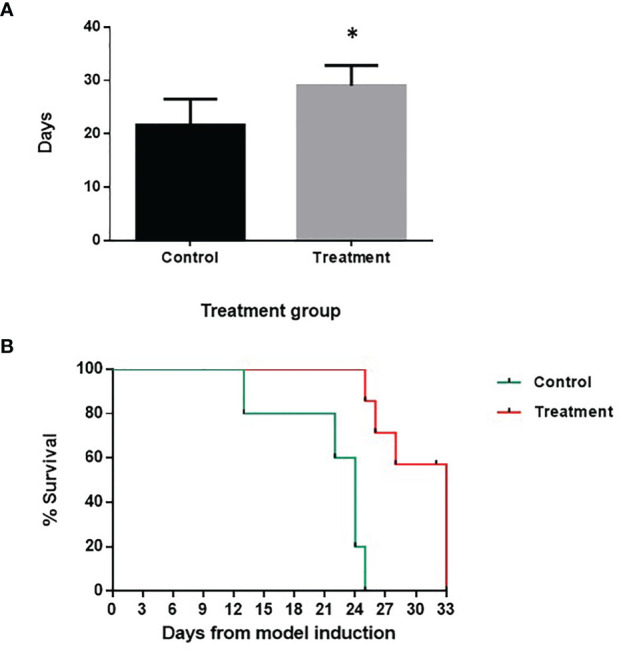
Effect of treatment on animal survival in the 4T1 breast cancer lung metastasis model. Treatment cycles started at day 13 after cell inoculation. **(A)** Median survival time (days) of control *vs*. treatment group. **(B)** Kaplan-Meier survival curve. There was a significant difference in the survival rate between the two groups. The statistical analysis was performed using the log-rank test, *p*-value <0.005 (*).

The median survival time of the control mice was 21.6 ± 4.9 days and 29 ± 3.8 days for the treatment group (*p*-value 0.029) (**Figure 9A**). On day 33, 40% of the animals in the treated group were still alive while all mice in the control group have died (**Figure 9B**). Kaplan-Meier analysis demonstrates a significant improvement in survival after treatment compared to the control group (*p*-value <0.005).

## Discussion

To preferentially target malignant cells, we have developed a novel approach that causes sub-ablative thermal damage to cancer cells to treat the lung metastases of mice bearing 4T1 metastatic tumors. Through the analysis of biodistribution, safety, toxicity, efficacy, and survival, we provide here an understanding of the effects of SaNPs and the Sarah Nanotechnology treatment on BALB/c mice.

Sarah Nanotechnology is a systemic treatment where the SaNPs are administered IV and localize on cancer cells through the use of the EPR effect that has attracted great interest, in conjunction with the further development of nanomedicine, enabling nanoparticle delivery and increased retention of the SaNPs in tumor tissues. Because of the SaNPs’ unique properties, in tumor and tumor metastases larger than 200 microns, the EPR effect is expected to be significant and the accumulation of SaNPs in the target tumor metastases will be higher than in non-tumor tissue, accordingly (25).

SaNPs have an amorphic structure and binding capability that facilitate surface attachment to the cancer cells and interaction with the cell membrane. Nanoparticle size, shape, surface functionalization, and concentration have been claimed to contribute to *in vivo* distribution and toxicity outcomes. Studies in mice through IV injection, examining the distribution and/or toxicity of various nanoparticles, have shown dose-dependence toxicity as well as significant accumulation in organs such as the liver and spleen. More specifically, small nanoparticles (10 nm) were shown to have a widespread distribution and found in various organs including the liver, spleen, kidney, heart, lungs, testis, brain, and thymus, whereas larger NPs (~250 nm) were mostly restricted to the liver and spleen, similar to SaNPs (135 nm) (26).

Our data showed that systemic administration of single as well as 3 repeated doses of SaNPs followed by AMF application to BALB/c mice was not associated with any significant adverse reactions nor any noticeable side effects or target organ toxicity. In particular, no necrosis was associated with the presence of SaNPs, and the observations in the liver and lungs were always sporadic and of minimal degree. The absence of inflammation and adverse reactions suggest that the SaNPs do not cause any toxicity or thermal damage, even after 3 repeated doses of SaNPs. Of note, there was a time- related recovery in the lungs and no progressive changes were observed in the liver after 25 weeks post treatment. These findings demonstrate that SaNPs at a maximal feasible dose in mice followed by AMF application, have no significant clinical or humoral acute or chronic toxicologic effects after systemic exposure.

The biodistribution studies showed that the optimal time for AMF application after IV injection of SaNP was 8hrs, at this timepoint SaNP accumulation in the lung metastases, was the highest and correlated with a low blood concentration. Animal studies with other nanoparticles have shown that immediately following IV injection, nanoparticles interact with blood components such as proteins, membranes, cells, and DNA, leading to the formation of a protein ‘corona’ on the nanoparticles thereby directing them to their ultimate sites of deposition, largely through the fixed macrophages in the liver and B cells in the spleen that are part of the MPS (27). In line with these findings, the percentage of injected SaNP that reaches the lung metastases increases over time and this phenomenon correlates with an inverse relationship that causes a decrease in the SaNP concentration in the blood. This is further supported by Maeda et al., that have claimed that the drug concentration in a tumor compared to that of the blood (T/B ratio) can be usually as high as 10–30 times, due to the EPR effect (28).

Notably, a comprehensive analysis of nanoparticle delivery to tumor cells has shown that a median of 0.7% of the injected dose (ID) can reach the tumor (29). This value was derived from 232 data sets. The median delivery efficiency has not improved in the past 10 years, suggesting that only 7 out of 1000 administered nanoparticles can actually enter a solid tumor in a mouse model. Our biodistribution studies show that the percentage of SaNPs left in the lungs after 4 weeks was ~0.35%, half of the median amount that reaches tumor cells, implying effective delivery of SaNPs to the target cells. We can assume that a small number of nanoparticles is seemingly sufficient to produce substantial, local heating leading to treatment efficacy, as supported by our data. Moreover, nanoparticles composed of inorganic materials, such as SaNPs, tend to provide a higher delivery efficiency than those made from organic materials (0.8 and 0.6% ID, respectively) (29). Sarah Nanotechnology offers high nanoparticle delivery efficiency because of its ability to accumulate at higher concentrations in the tumor tissue, with no toxicity to other organs.

As underlined by our data, in the long-term biodistribution evaluation, the majority of SaNPs were accumulated in the liver, probably due to uptake by the resident macrophages (i.e., Kupffer cells) (30). Moreover, resident macrophages of the lungs and spleen contribute to substantial particle uptake as supported by the results of the safety study and histopathology of these organs. This is consistent with the observations of other studies (31, 32). This finding is of great importance as it determines time intervals between Sarah Nanotechnology treatment schedule in patients with solid tumors receiving a chronic therapeutic regimen. However, further assessments are required in other animal models.

Our proof-of-concept breast cancer metastatic animal model studies demonstrated that Sarah Nanotechnology treatment was able to significantly inhibit tumor progression and prolong the survival of BALB/c bearing 4T1 metastatic tumors, that were treated with repeated cycles of Sarah Nanotechnology treatment. The results of several efficacy studies, showed a significant reduction in the number of metastases as well as in their size and viability, in the treated compared to the control mice following 3 treatment cycles of Sarah Nanotechnology.

Furthermore, the survival of treated mice was significantly improved compared to the control. Due to its highly aggressive nature, only few studies examining survival using the 4T1 metastatic model have been published (33). Of note, in a study conducted by Purwanti and colleagues examining the survival of 4T1 tumor bearing BALB/c mice, inoculated with 1x10^4^ 4T1 cells, mice started to die at day 17, and the median survival time was 26.2, 26.8, 25.6, and 27.6 days for the control and the treatment groups, respectively (34). There was no statistically significant difference among the groups in the survival rate. Therefore, our results are encouraging as reducing tumor burden and eventually prolonging the survival of animals bearing lung metastatic tumors has previously proven to be a challenging task.

The presented magnetic hyperthermia nanoplatform is a physical approach that is not limited by the development of resistance to treatments that occurs with all the present known systemic antineoplastic approaches (35). The lack of development of resistance to thermal therapy of tumor cells, as opposed to intrinsic or acquired drug resistance, and the low toxicity of the approach in mice suggests that this technology could be used as a tool to chronically treat advanced cancer without causing side effects. Furthermore, while chemotherapeutic agents lack specificity, the engineered SaNPs may provide a good choice for effectively overcoming the drawbacks of traditional materials in biomedical therapy, such as multidrug resistance of cancer cells (36–38), due to their unique physicochemical characteristics and the fact that they are activated only when exposed to a magnetic field.

## Conclusion

Our results demonstrate that Sarah Nanotechnology treatment, exhibits high specificity and efficiency in damaging and destroying metastatic cancer cells. The studies presented herein prove the feasibility and effectiveness of our approach in treating this type of cancer in BALB/c mice *in vivo*. In addition, they provide a solid basis for the development of Sarah Nanotechnology as a novel approach and therapeutic strategy for clinical application in treating metastatic solid tumors following the failure of all standard treatments.

## Data Availability Statement

The raw data supporting the conclusions of this article will be made available by the authors, without undue reservation.

## Ethics Statement

The animal study was reviewed and approved by Bar Ilan University, Israel.

## Author Contributions

Conceptualization, SK, RKD, and RH. Methodology, SK, RKD, PR, AA, RK, and MC-E. Formal analysis, SK, RKD, and AN. Investigation, SK, ES, and RR. Writing – original draft preparation, SK. Writing – review and editing, SK, RR, OS, and AC. Visualization, ES and RR. Supervision, OS, GG, and AC. Project administration, RH, and OS. All authors contributed to the article and approved the submitted version.

## Funding

This work was funded by New Phase Ltd.

## Conflict of Interest

Authors SK, RKD, RH, ES, ME, PR, RR, RK, AA, OS, and MC-E were employed by New Phase Ltd.

The remaining authors declare that the research was conducted in the absence of any commercial or financial relationships that could be construed as a potential conflict of interest.

The authors declare that this study received funding from New Phase Ltd. The funder had the following involvement with the study: Study design, collection, analysis, interpretation of data, and the writing of this article.

## Publisher’s Note

All claims expressed in this article are solely those of the authors and do not necessarily represent those of their affiliated organizations, or those of the publisher, the editors and the reviewers. Any product that may be evaluated in this article, or claim that may be made by its manufacturer, is not guaranteed or endorsed by the publisher.
